# (20S) Ginsenoside Rh2 Exerts Its Anti-Tumor Effect by Disrupting the HSP90A-Cdc37 System in Human Liver Cancer Cells

**DOI:** 10.3390/ijms222313170

**Published:** 2021-12-06

**Authors:** Chen Chen, Yu-Shi Wang, En-Ting Zhang, Gang-Ao Li, Wen-Yuan Liu, Yang Li, Ying-Hua Jin

**Affiliations:** Key Laboratory for Molecular Enzymology and Engineering of the Ministry of Education, School of Life Sciences, Jilin University, Changchun 130012, China; cchen16@mails.jlu.edu.cn (C.C.); wangyushi0317@hotmail.com (Y.-S.W.); zhanget19@mails.jlu.edu.cn (E.-T.Z.); Lga1997@163.com (G.-A.L.); lwy9977520@163.com (W.-Y.L.); liyang915@jlu.edu.cn (Y.L.)

**Keywords:** (20S) G-Rh2, HSP90A, Cdc37, phage display, proteasome degradation pathway, autophagy, human liver cancer

## Abstract

(20S) ginsenoside Rh2 (G-Rh2), a major bioactive metabolite of ginseng, effectively inhibits the survival and proliferation of human liver cancer cells. However, its molecular targets and working mechanism remain largely unknown. Excitingly, we screened out heat shock protein 90 alpha (HSP90A), a key regulatory protein associated with liver cancer, as a potential target of (20S) G-Rh2 by phage display analysis and mass spectrometry. The molecular docking and thermal shift analyses demonstrated that (20S) G-Rh2 directly bound to HSP90A, and this binding was confirmed to inhibit the interaction between HSP90A and its co-chaperone, cell division cycle control protein 37 (Cdc37). It is well-known that the HSP90A-Cdc37 system aids in the folding and maturation of cyclin-dependent kinases (CDKs). As expected, CDK4 and CDK6, the two G_0_-G_1_ phase promoting kinases as well as CDK2, a key G_1_-S phase transition promoting kinase, were significantly downregulated with (20S) G-Rh2 treatment, and these downregulations were mediated by the proteasome pathway. In the same condition, the cell cycle was arrested at the G_0_-G_1_ phase and cell growth was inhibited significantly by (20S) G-Rh2 treatment. Taken together, this study for the first time reveals that (20S) G-Rh2 exerts its anti-tumor effect by targeting HSP90A and consequently disturbing the HSP90A-Cdc37 chaperone system. HSP90A is frequently overexpressed in human hepatoma cells and the higher expression is closely correlated to the poor prognosis of liver cancer patients. Thus, (20S) G-Rh2 might become a promising alternative drug for liver cancer therapy.

## 1. Introduction

Heat shock protein 90 (Hsp90) is a well-known adenosine 5′-triphosphate (ATP)-dependent protein chaperone that facilitates the maturation of several hundred protein substrates including protein kinases, steroid hormone receptors, ribonucleoproteins, and transcription factors [1,2,3,4]. There are two Hsp90 genes in *Homo sapiens*, *HSP90AA1* and *HSP90AB1*, encoding cytosolic proteins HSP90A and HSP90B, respectively [5,6,7]. Although there have been many studies on Hsp90, the functional difference between HSP90A and HSP90B is still not clear. A large number of co-chaperones regulate the conformational changes of the Hsp90 homodimer through a dynamic process that includes many protein–protein interactions (PPI). Different co-chaperones including Hsp70, Hsp40, Hsp70/Hsp90 organizing protein (HOP), cell division cycle 37 (Cdc37), in concert with Hsp90 to form transient complexes not only mediate the Hsp90 ATPase cycle but also direct a broad range of specific clients to Hsp90 [8]. Both Hsp90 and Hsp70 are critical in the regulation of protein folding. At the same time, the post-translational modifications of Hsp90 and Hsp70 affect their ATPase activity, localization, and client binding capability to influence a range of biological processes [9,10]. Additionally, Cdc37, also known as a cell division cycle control protein, is one of the best-studied co-chaperones of Hsp90 and is assumed to be involved in the regulation of various protein kinases including CDKs, eukaryotic translation initiation factor 2 alpha kinase 1 (EIF2AK1), and AKT serine/threonine kinase 1 (AKT1) [11]. The major function of Cdc37 is to selectively recognize and combine unfolded kinase clients, load them onto Hsp90, and form an Hsp90-Cdc37-kinase complex for further maturation [12,13,14]. Hsp90 is often overexpressed in various cancers in response to the hypoxic, acidotic, and nutrient-deprived tumor environment [15,16]. The higher expression of Hsp90 might be involved in establishing an immunosuppressive tumor environment, which further leads to tumor formation, poor prognosis, and therapeutic resistance. Furthermore, cancer progression is accompanied by the activation and overexpression of oncoproteins that require Hsp90 to fold and stabilize [17]. Therefore, it is critical to control the expression and activity of Hsp90 to prevent them from promoting tumor formation and further malignant growth, and targeting Hsp90 is a compelling direction for cancer diagnostics and therapies [18].

The major protein degradation systems in eukaryotic cells include the ubiquitin-proteasome system (UPS) and autophagy-lysosome pathway (ALP). UPS substrates are covalently conjugated with one or more ubiquitin tags and then targeted to 26S proteasome for proteolysis into small peptides [19,20]. Compared with UPS, the autophagy process requires formation of a double-membrane autophagosome fused with lysosome to accomplish the substrates degradation [21,22,23]. The degradation pathway choice is mainly determined by the substrate size. UPS mostly degrades single, unfolded polypeptides, whereas autophagy primarily deals with larger, cytosolic structures [24]. Proteostasis is a key requirement for cell metabolism and organelle biogenesis to maintain cellular homeostasis in response to stress stimuli.

Liver cancer is the fifth most common malignant tumor in the world and ranks as the third leading cause of cancer-related death. The latest epidemiological survey report showed that there are more than 900,000 new liver cancer cases worldwide each year, and nearly half of them occur in China [25,26]. Deregulated cell proliferation is a key hallmark of cancer, and a majority of anti-cancer drugs are developed to target cell cycle-regulating proteins directly or indirectly at present [27].

Ginsenoside Rh2, with a dammarane skeleton, has been identified as a major bioactive constituent of ginseng to inhibit the viability of various cancer cell lines [28,29]. Depending on the chiral structure of carbon-20, ginsenoside Rh2 can be divided into S-type and R-type configurations. Compared with (20R) G-Rh2, (20S) G-Rh2 has a more prominent growth inhibitory effect on cancer cells. Meanwhile, the cytotoxic effect of (20S) G-Rh2 on normal tissue cells is much weaker than that on tumor tissue cells [30]. Previous studies have shown that (20S) G-Rh2 arrested the cell cycle at G_1_ phase by downregulating Cyclin E-CDK2 activity and suppressed the proliferation of SK-HEP-1 cells [31]. However, the exact target of (20S) G-Rh2, which causes cell cycle arrest has not yet been identified. Our current study aimed to elucidate the underlying mechanism of (20S) G-Rh2-induced cell growth inhibition in liver cancer cells by identifying its critical targets.

## 2. Results

### 2.1. HSP90AA1 May Serve as a Critical Liver Cancer-Related Gene Targeted by (20S) G-Rh2

**Figure 1:**
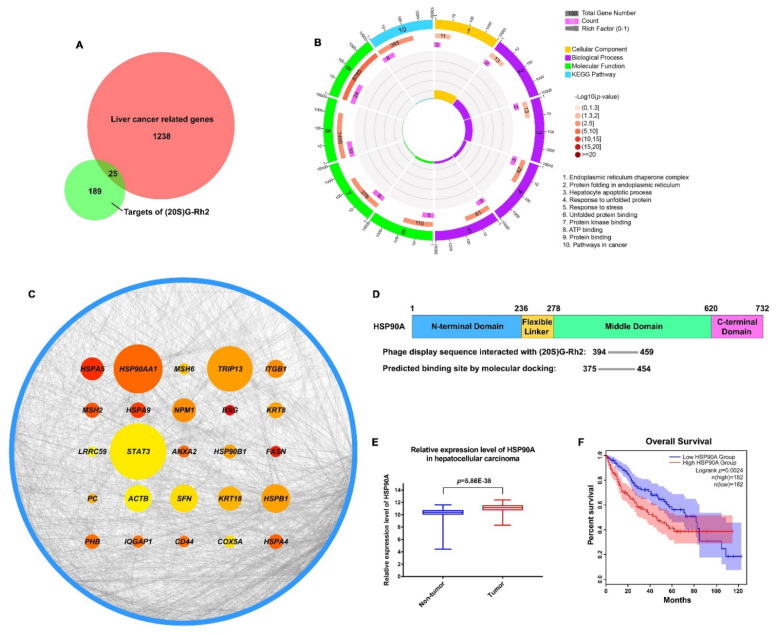
Screening for liver cancer-related genes targeted by (20S) G-Rh2. (**A**) The Venn diagram of liver cancer-related genes and targets of (20S) G-Rh2. The red part represents liver cancer-related genes, and the green part represents targets of (20S) G-Rh2. (**B**) Gene Ontology (GO) and KEGG pathway enrichment analyses of the overlapped genes. (**C**) Network topology analysis of the overlapped genes. The node size is proportional to the number of neighbor genes interacting with these 25 targets in the background network of the human genome, and the blue circle represents their neighbor genes. The node color represents the propensity to bind (20S) G-Rh2, and the closer to red, the more inclined to bind (20S) G-Rh2. (**D**) Graphical view of the binding location of (20S) G-Rh2 on HSP90A. (**Top**) The functional domains of HSP90A. (**Bottom**) The phage display sequence of HSP90A interacted with (20S) G-Rh2 and the predicted binding site by molecular docking. (**E**) Relative expression level of HSP90A in hepatocellular carcinoma based on the GSE14520 dataset in GEO database. (**F**) Survival analysis of HSP90A in hepatocellular carcinoma based on the TCGA database using GEPIA2. The shadow areas represent a 95% confidence interval.

In order to find the potential targets of (20S) G-Rh2 in liver cancer, we first used amino polyethylene glycol-polyacrylamide copolymer (PEGA) resin covalently conjugated with (20S) G-Rh2 or (20R) G-Rh2 to enrich proteins from whole-cell lysate of HepG2 cells. Then, we identified the enriched proteins by mass spectrometry and found 214 potential targets that were more inclined to bind (20S) G-Rh2 (Figure 1A). To further investigate the pharmacological mechanism of (20S) G-Rh2 against liver cancer, 1263 related genes of hepatocellular carcinoma were obtained from the GeneCards database [32] and DisGeNET database [33]. The Venn diagram analysis showed that (20S) G-Rh2 shared 25 putative targets with the liver cancer-related genes (Figure 1A and Table S1). Thus, further functional analysis of these 25 potential targets of (20S) G-Rh2 might help us understand the anti-tumor mechanism of (20S) G-Rh2 in liver cancer. Next, we performed the Gene Ontology (GO) and Kyoto Encyclopedia of Genes and Genomes (KEGG) pathway enrichment analysis of these 25 genes using Database for Annotation, Visualization, and Integrated Discovery (DAVID) [34]. The top enrichments in molecular function, cellular component, biological process, and KEGG pathway categories were related to endoplasmic reticulum (ER) stress and protein folding process (Figure 1B). Network topology analysis of these 25 genes was performed to find out the key target by setting all human genes as the background. As shown in Figure 1C and Table S1, we found that *HSP90AA1* played a key role in the human gene network associated with liver cancer, and HSP90A (encoded by *HSP90AA1*) was more likely to bind (20S) G-Rh2. Interestingly, our previous phage display study also showed that the middle domain of HSP90A bonded to the (20S) G-Rh2-PEGA resin, but not the (20R) G-Rh2-PEGA resin (Figure 1D) [35,36], which once again raised the credibility of HSP90A as a target for (20S) G-Rh2. Importantly, it was observed that the expression level of HSP90A was significantly elevated in liver cancer tissues compared with normal tissues based on the GSE14520 dataset in the Gene Expression Omnibus (GEO) database [37] (Figure 1E). Moreover, the survival analysis in Figure 1F revealed that the increased level of HSP90A was strongly associated with poor overall survival based on the Cancer Genome Atlas (TCGA) database [38] using Gene Expression Profiling Interactive Analysis (GEPIA2) [39]. Therefore, it is reasonable to focus on HSP90A as a potential target of (20S) G-Rh2, which may help us elucidate the anti-tumor mechanism of (20S) G-Rh2 in human liver cancer.

### 2.2. Binding Modes of HSP90A and (20S) G-Rh2 Were Predicted by Molecular Docking

**Figure 2:**
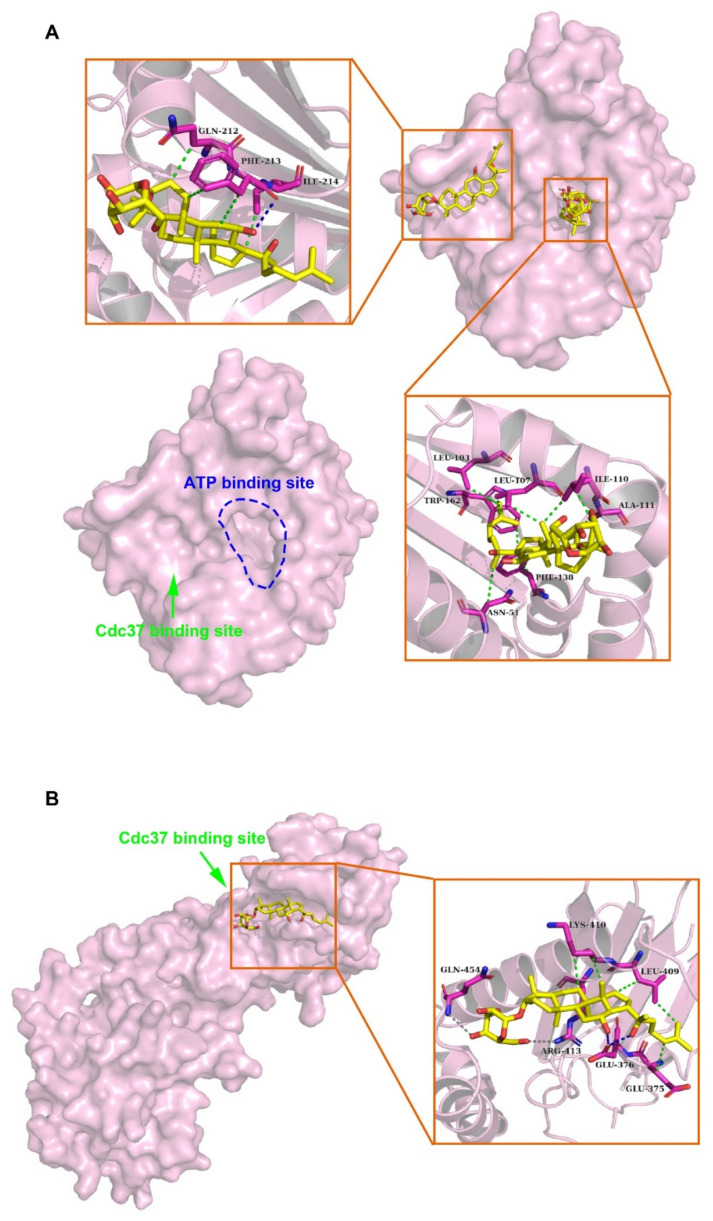
Molecular mechanisms of interactions between HSP90A and (20S) G-Rh2. (**A**) Molecular docking illustrations show two potential binding sites of (20S) G-Rh2 on HSP90A N-terminal domain (HSP90A_N) and the detailed amino acid residues in these two binding regions. (**B**) Potential binding site analysis of (20S) G-Rh2 on HSP90A MC domain (HSP90A_MC) and the surrounding amino acid residues by molecular docking. Overall structure with the surface of HSP90A is colored magenta. Insets are the detailed binding regions of (20S) G-Rh2 on HSP90A. HSP90A is represented as a magenta cartoon, and (20S) G-Rh2 is shown as yellow sticks. The detailed residues are highlighted within the stick model. Hydrophobic interactions, electrostatic interactions, and hydrogen bonds are displayed as green dotted lines, gray dotted lines, and blue dotted lines, respectively.

Molecular docking was performed to predict the interaction between HSP90A and (20S) G-Rh2. Due to the lack of structure analysis for the full-length HSP90A, we used the structure files for the N-terminal domain of HSP90A (HSP90A_N, PDB ID: 4BQG), and the middle and C-terminal domain of HSP90A (HSP90A_MC, PDB ID: 3Q6M), respectively to perform the docking analysis. Each docking result by AutoDock tools (version 4.2.6) presented 10 different conformations, and we selected the conformations with free energy of binding less than –5 kcal/mol for further analysis (Table 1). The optimum binding site for (20S) G-Rh2 on HSP90A_N overlapped with the ATP binding site of HSP90A [40], indicating that (20S) G-Rh2 might affect the folding of HSP90A client proteins. Additionally, the result showed that HSP90A_N interacted with (20S) G-Rh2 mainly by hydrophobic interactions (with N51, L103, L107, I110, A111, F138, and W162) (Figure 2A). Another binding site for (20S) G-Rh2 on HSP90A_N was located in the Cdc37 binding site [41], indicating that (20S) G-Rh2 might disrupt the interaction between HSP90A and Cdc37. Similarly, the molecular forces of this binding site were mainly hydrophobic interactions (with Q212, F213, and I214) (Figure 2A). The docking analysis of HSP90A_MC with (20S) G-Rh2 showed that (20S) G-Rh2 might occupy another binding site between HSP90A and Cdc37 [14]. Unlike the interaction with HSP90A_N, the molecular forces of this binding site included electrostatic interactions (with R413 and Q454) and some hydrophobic interactions (with E375, L409, and K410) (Figure 2B). Taken together, (20S) G-Rh2 may interact with both N-terminal and middle domains of HSP90A, thus disrupting the interaction between HSP90A and Cdc37.

### 2.3. (20S) G-Rh2 Interacted with HSP90A In Vitro

**Figure 3:**
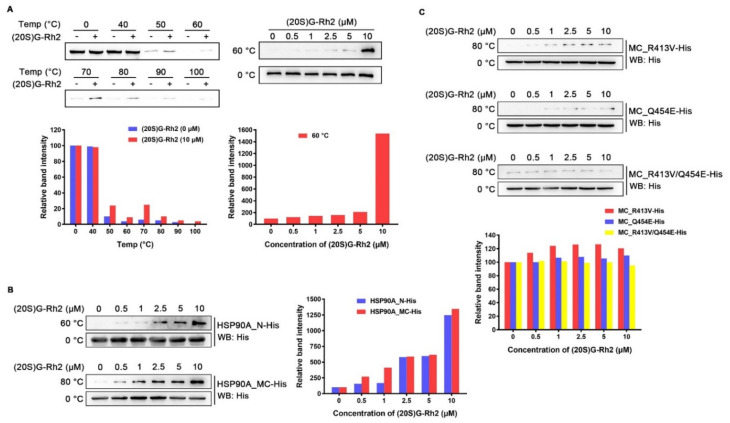
Interaction between HSP90A and (20S) G-Rh2. (**A**, **Left**) Cellular thermal shift assay was performed to investigate the thermal stability changes of endogenous HSP90A under treatment with 10 μM (20S) G-Rh2 at the indicated temperatures. (**Right**) The thermal stability changes of endogenous HSP90A under treatment with different concentrations of (20S) G-Rh2 at 60 °C by cellular iso-thermal dose response assay. (**Bottom**) Quantitative analyses of CETSA and ITDR. (**B**, **Left**) Iso-thermal dose response assay in vitro was performed to investigate the thermal stability changes of HSP90A_N and HSP90A_MC under treatment with different concentrations of (20S) G-Rh2 at the indicated temperatures. (**Right**) Quantitative analyses of ITDR in vitro. (**C**, **Top**) The thermal stability changes of HSP90A_MC-R413V, HSP90A_MC-Q454E, and HSP90A_MC-R413V/Q454E treated with different concentrations of (20S) G-Rh2 in vitro by iso-thermal dose response assay. (**Bottom**) Quantitative analyses of ITDR in vitro.

To examine whether (20S) G-Rh2 can interact with intracellular HSP90A, we performed cellular thermal shift assay (CETSA) in HepG2 cells. The result demonstrated that (20S) G-Rh2 can penetrate into cells and bind to intracellular HSP90A, and significantly increase the thermal stability of full-length HSP90A (Figure 3A). Because molecular docking analysis showed that (20S) G-Rh2 may interact with both the N-terminal domain and the middle and C-terminal domain of HSP90A, we constructed the MC domain truncated HSP90A (HSP90A_N with residues 1-236 retained) and the N-terminal domain truncated HSP90A (HSP90A_MC with residues 293-732 retained) with C-terminal His tag, respectively. The in vitro expression of HSP90A_N and HSP90A_MC for the iso-thermal dose response (ITDR) assay can eliminate the effect of other cellular components on the result, and the heating temperature for the ITDR assay was selected according to the protein melting temperature (T_m_). As shown in Figure 3B, (20S) G-Rh2 enhanced the thermal stability of HSP90A_N and HSP90A_MC, respectively, in a dose-dependent manner, indicating that (20S) G-Rh2 bound to both the N-terminal domain and the MC domain of HSP90A. The docking result showed that the interactions of (20S) G-Rh2 with the N-terminal domain were mainly hydrophobic interactions including two potential binding sites (Figure 2A), and this made the selection of mutation sites much more difficult because hydrophobic interactions are much weaker than other molecular forces. The substitution of residues cannot effectively change the hydrophobic interactions, and the protein structure may be severely affected if all the surrounding residues are mutated. However, there were electrostatic interactions between (20S) G-Rh2 and the MC domain of HSP90A. Thus, we mutated two residues with potential electrostatic interactions (Figure 2B), and determined the thermal stability changes of the MC domain by ITDR in vitro. Even under 10 μM (20S) G-Rh2 treatment, the thermal stability of MC_R413V and MC_Q454E was not obviously enhanced. Furthermore, there was no change in the thermal stability of MC_R413V/Q454E (Figure 3C), indicating that R413 and Q454 are critical residues for the interaction of HSP90A and (20S) G-Rh2, as predicted by molecular docking.

### 2.4. (20S) G-Rh2 Induced CDKs Degradation and Cell Cycle Arrest by Disrupting the Interaction between HSP90A and Cdc37

**Figure 4:**
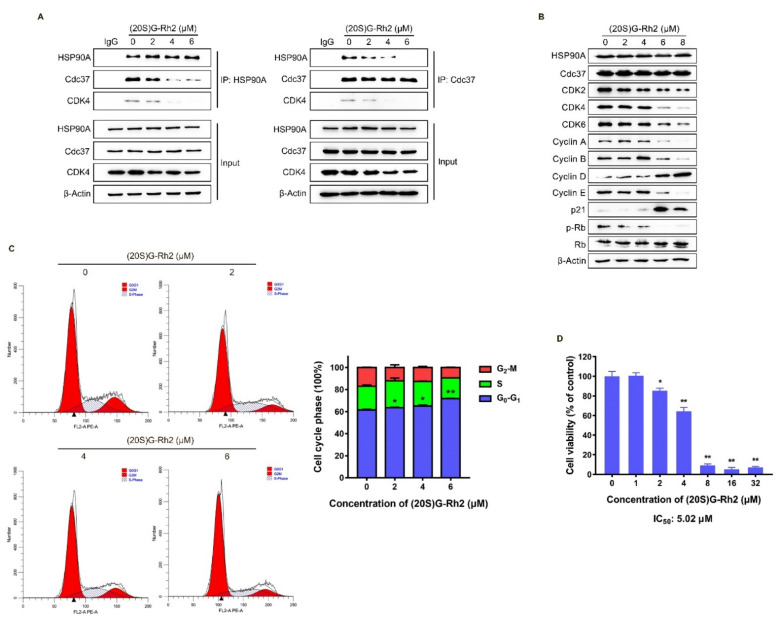
(20S) G-Rh2-induced interaction disruption of HSP90A and Cdc37, degradation of CDKs, cell cycle arrest, and proliferation inhibition of HepG2 cells. (**A**) Immunoprecipitation was performed with whole-cell lysate under treatment with different concentrations of (20S) G-Rh2 for 24 h, and the interaction was analyzed by an immunoblot. (**B**) Immunoblotting analysis of the cell cycle-related protein levels in HepG2 cells treated with different concentrations of (20S) G-Rh2 for 24 h. (**C**, **Left**) Cell cycle distribution was determined by PI staining of HepG2 cells after treatment with the indicated concentrations of (20S) G-Rh2 for 24 h. (**Right**) The cell cycle distribution is represented as a graphic histogram. (**D**) HepG2 cells were treated with the indicated concentrations of (20S) G-Rh2 for 48 h, and the cell viability was determined by MTT assay. All data are shown as the mean ± SD of experiments performed in triplicate. A two-tail Student’s *t*-test was used for statistical analyses (* *p* < 0.01, ** *p* < 0.001).

The above molecular docking results showed that the binding site of (20S) G-Rh2 on HSP90A was located near the Cdc37 binding site (Figure 2A,B), indicating that (20S) G-Rh2 may inhibit the interaction between HSP90A and Cdc37. Thus, we selected HepG2 cells with weak migration capability and SK-HEP-1 cells with strong migration capability for the following experiments to verify this prediction. Co-immunoprecipitation (Co-IP) experiments were performed in HepG2 cells and SK-HEP-1 cells treated with increased concentrations of (20S) G-Rh2. The results demonstrated that (20S) G-Rh2 significantly attenuated the interaction between HSP90A and Cdc37 in a dose-dependent manner (Figure 4A and Figure S1A). As we know, co-chaperone Cdc37 is responsible for recruiting kinases to Hsp90, and it has been assumed to be a specifically kinase-targeting subunit for the Hsp90 chaperone system [42]. Additionally, CDK4, one of the most well-studied kinase clients of Hsp90-Cdc37 [14], was determined by immunoblot to confirm the influence of (20S) G-Rh2 on the interaction between HSP90A and Cdc37. As shown in Figure 4A, (20S) G-Rh2 also disrupted the interaction of CDK4 and HSP90A-Cdc37 system. It was reported that the correct folding of CDK4 is highly correlated with Hsp90-Cdc37, and plays a key role in the cell cycle progression from G_0_ to G_1_. Thus, we determined the protein levels of major regulators in cell cycle after (20S) G-Rh2 treatment using an immunoblot. Detectable decreases in CDK2, CDK4, CDK6, Cyclin A, Cyclin B, Cyclin E, and p-Rb were observed in HepG2 cells, while the levels of Cyclin D and p21 were significantly upregulated in a dose-dependent manner (Figure 4B). Furthermore, we also observed a reduced level of CDK4 in the input of Figure 4A. Similarly, a significant decrease in CDKs was also observed in SK-HEP-1 cells treated with (20S) G-Rh2 (Figure S1B). We next performed a cell cycle distribution analysis by staining DNA with propidium iodide (PI) using flow cytometry, and a G_0_–G_1_ phase cell cycle arrest was observed in HepG2 cells after treatment with different concentrations of (20S) G-Rh2 for 24 h (Figure 4C), and (20S) G-Rh2-induced G_1_ phase arrest of SK-HEP-1 cells has been reported in the previous study [31]. At the same time, we also found that (20S) G-Rh2 significantly inhibited the viability of HepG2 cells and SK-HEP-1 cells by methyl thiazolyl tetrazolium (MTT) assays (Figure 4D and Figure S1C). Similar to the regulatory mechanism of CDK4, the correct folding and maturation of CDK2 and CDK6 also require the participation of the Hsp90-Cdc37 system [43,44]. Therefore, we suggested that (20S) G-Rh2 inhibited the maturation of CDKs by disrupting the interaction between HSP90A and Cdc37, leading to the degradation of CDKs.

### 2.5. (20S) G-Rh2 Inhibited CDKs Maturation and Induced Proteasome Degradation of CDKs

**Figure 5:**
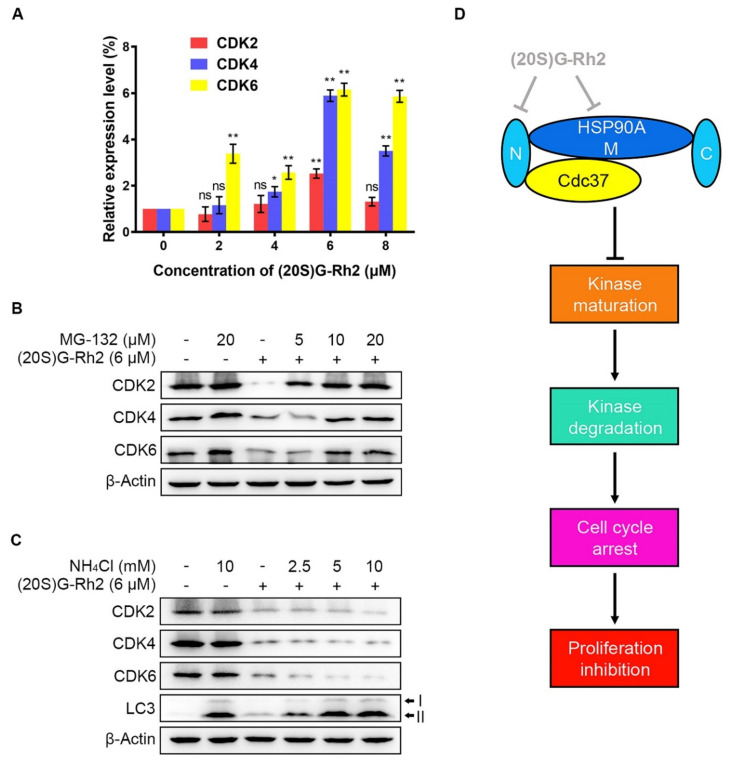
(20S) G-Rh2-induced proteasome degradation of CDKs. (**A**) Relative gene expression levels of CDKs in HepG2 cells were determined by qRT-PCR under treatment with the indicated concentrations of (20S) G-Rh2 for 24 h. Data are shown as the mean ± SD of experiments performed in triplicate. A two-tail Student’s *t*-test was used for statistical analyses (* *p* < 0.01, ** *p* < 0.001). (**B**) HepG2 cells were pre-treated with different concentrations of MG-132 for 4 h before treatment with (+) or without (−) 6 μM (20S) G-Rh2 for 24 h, and the protein levels of CDKs were determined by an immunoblot. (**C**) HepG2 cells were treated with different concentrations of NH_4_Cl with (+) or without (−) the presence of (20S) G-Rh2 (6 μM) for 24 h, and the protein levels of CDKs were determined by an immunoblot. (**D**) Schematic model of the process in which (20S) G-Rh2 modulates the HSP90A-Cdc37 chaperone cycle. (20S) G-Rh2 inhibited the HSP90A-Cdc37 PPI through binding to the N-terminal domain and the middle domain of HSP90A, inhibiting the maturation of CDKs to induce their degradation and the proliferation inhibition of HepG2 cells. ns: not significant.

Due to a detectable decrease in protein levels possibly caused by a protein degradation or a decrease in mRNA level, quantitative real-time polymerase chain reaction (qRT-PCR) was performed to determine the alteration in gene expression levels of CDKs after treatment of different concentrations of (20S) G-Rh2. We did not observe a significant decrease in the mRNA levels of CDKs. In contrast, the gene expression levels of CDKs in HepG2 cells were evidently upregulated with the elevated concentrations of (20S) G-Rh2 (Figure 5A). Given that there are two major pathways for misfolded protein degradation—ubiquitin-proteasome system (UPS) and autophagy-lysosome pathway (ALP) [24]—we next investigated which degradation pathway the decrease in CDKs protein levels under treatment with (20S) G-Rh2 was mediated by. The result showed that MG-132, a specific proteasome inhibitor, significantly inhibited CDKs degradation induced by (20S) G-Rh2 in a dose-dependent manner (Figure 5B). However, the decrease in CDKs induced by (20S) G-Rh2 could not be reversed under treatment with the gradient concentrations of ammonium chloride (NH_4_Cl), which was used as a lysosome inhibitor (Figure 5C). Additionally, we observed a significant accumulation of LC3-II protein level with the increased concentrations of NH_4_Cl in HepG2 cells, suggesting that NH_4_Cl blocked the autophagy degradation pathway by inhibiting lysosome activity (Figure 5C). In conclusion, the above results demonstrated that UPS, but not ALP, mediated the degradation of CDKs in HepG2 cells induced by (20S) G-Rh2.

## 3. Discussion

The previous studies showed that (20S) G-Rh2 displayed an inhibitory effect on cancer development, progression, and metastasis by triggering the G_1_ phase arrest and inducing the endogenous apoptosis in liver cancer cells [31,45]. However, the exact cellular targets and the underlying mechanism remained unclear. In this study, we identified HSP90A as an important target of (20S) G-Rh2 in liver cancer cells, and for the first time, we elucidated the anti-tumor mechanism of (20S) G-Rh2 inducing G_1_ phase arrest by targeting and disrupting the HSP90A-Cdc37 system. We first synthesized PEGA resin covalently conjugated with (20S) G-Rh2 to enrich proteins from whole-cell lysate of HepG2 cells, and identified 214 potential targets of (20S) G-Rh2 using mass spectrometry analysis. Then, we obtained 25 putative genes shared with liver cancer based on the GeneCards database and DisGeNET database. Enrichment analysis and network topology analysis of these 25 genes were then performed, and we selected *HSP90AA1* for further study because it plays a pivotal role in the gene network associated with liver cancer and preferred to bind to (20S) G-Rh2. As we know, most proteins in cells exist in the form of a protein complex. These 214 identified proteins can only be regarded as potential targets of (20S) G-Rh2, as they may be enriched from cell lysate through direct or indirect interactions with (20S) G-Rh2. Thus, further interaction and function experiments are still needed to validate their reliabilities. Then, we predicted the binding modes of HSP90A and (20S) G-Rh2 using molecular docking. It was shown that the potential binding sites for (20S) G-Rh2 on HSP90A was located at the Cdc37 binding site and ATP binding site, indicating that (20S) G-Rh2 may affect the correct folding of client proteins by disrupting the interaction between HSP90A and Cdc37. We used the N-terminal domain and the MC domain of HSP90A to perform docking analysis, respectively, because we did not find a crystal structure for the full-length HSP90A in available studies. It was reported that ATP binding results in two different states of the Hsp90-Cdc37 PPI. Without ATP binding, the Hsp90 dimer remains in an open conformation and binds to Cdc37 via its N-terminal domain, limiting its ATPase. When ATP binds to Hsp90, Cdc37 moves to the middle domain of Hsp90 with a kinase client and relieves the inhibition of ATPase, inducing the Hsp90 dimer to adopt a closed conformation [14,41]. Our docking results showed that (20S) G-Rh2 may inhibit Cdc37 binding modes of HSP90A. Moreover, combined with our previous phage display analysis, the sequence located on the middle domain of HSP90A that bound to (20S) G-Rh2 overlapped with the predicted binding site by molecular docking and was also the same site interacting with Cdc37 (Figure 1D). In order to validate the above prediction and hypothesis, cellular thermal shift assay and iso-thermal dose response assay in vitro were performed, and the interaction between HSP90A and (20S) G-Rh2 was confirmed. We also found that R413 and Q454 at the middle domain of HSP90A were critical residues for this interaction.

It is known that the Hsp90 chaperone system helps in the proper folding and maturation of nascent peptide chains and misfolded proteins to prevent protein aggregation and degradation [46,47,48]. Research in recent years has identified numerous co-chaperones of Hsp90. Among them, Cdc37, a cell division cycle control protein, acts as a hunter in the Hsp90-Cdc37 system by specifically recognizing and binding to client kinases [11], suggesting that the disruption of this system by (20S) G-Rh2 may have a great effect on the cell cycle process. The Co-IP assays in HepG2 cells and SK-HEP-1 cells confirmed that (20S) G-Rh2 significantly attenuated the interaction between HSP90A and Cdc37 in a dose-dependent manner, which was consistent with the previous results of phage display, molecular docking, and ITDR. At the same time, we also observed that the protein levels of CDKs, Cyclins, and p-Rb decreased detectably, but p21 had a significant increase, and the cell cycle distribution showed a G_0_–G_1_ phase arrest when treated with (20S) G-Rh2. The specific Cyclin-CDK complexes act primarily as checkpoints for each phase to promote cell cycle progression, and highly expressed CDKs obviously lead to poor overall survivals of cancer patients [49,50]. Cyclin-dependent kinase inhibitor 1A, also known as p21, is primarily responsible for G_1_–S phase transition. It binds to and inhibits the activity of Cyclin E/A-CDK2 complexes. Cyclin D-CDK4/6 complexes are major kinases for Rb phosphorylation, and the phosphorylated Rb releases E2F to transcribe genes necessary for cell cycle progression [51,52]. It was the decrease in CDK4/6 that led to G_0_–G_1_ phase arrest in HepG2 cells under (20S) G-Rh2 treatment. Even though we observed an upregulation of Cyclin D, cell cycle arrest was still induced by (20S) G-Rh2 in HepG2 cells because of the significant decrease in CDK4/6. We suggested that the downregulation of CDKs was caused by their incorrect folding due to the disruption of HSP90A-Cdc37 system by (20S) G-Rh2. The qRT-PCR result demonstrated that (20S) G-Rh2 did not reduce the mRNA level of these CDKs, indicating that the decrease in CDKs was caused by protein degradation. As we know, there are two major misfolded protein degradation pathways in eukaryotic cells: ubiquitin-proteasome system (UPS) and autophagy-lysosome pathway (ALP). Thus, we used MG-132 (proteasome inhibitor) or NH_4_Cl (lysosome inhibitor), respectively, to investigate the degradation mechanism of CDKs. The immunoblot analyses showed that (20S) G-Rh2 induced the protein degradation of CDKs in HepG2 cells through UPS but not ALP. UPS and ALP have long been treated as two completely isolated systems. However, growing evidence shows that there are multiple connections between these two major degradation pathways. For example, ubiquitin no longer serves as a substrate specific label for the proteasome system, and both degradation pathways can recognize and degrade the ubiquitinated substrates [24,53]. Therefore, we did not further investigate the ubiquitination modifications of CDKs.

To date, more than thirty Hsp90 inhibitors have entered clinical trials, but further clinical safety and clinical validity trials have limited their applications due to multiple side effects [54,55]. Among them, TAS-116, an oral inhibitor of Hsp90, has been reported to enter the single-arm phase II study [56]. These inhibitors were discovered or designed to completely inhibit the function of Hsp90 by occupying the ATP pocket [57,58]. The complete inhibition of Hsp90 inevitably results in the incorrect folding of numerous client proteins, which may cause potential toxicities. (20S) G-Rh2, as a natural compound from ginseng, unlike other synthetic compounds, has a moderate affinity with HSP90A. The above docking and thermal shift results showed that HSP90A may have more than one (20S) G-Rh2 binding site, which may competitively inhibit the binding of ATP or Cdc37. Since HSP90A has an extensive range of co-chaperones and clients, the regulation of these proteins by (20S) G-Rh2 needs to be further investigated. Moreover, the phage display and mass spectrometry analyses also showed that (20S) G-Rh2 exerted its anti-tumor effect by targeting multiple proteins. Of course, in our previous studies, Annexin A2 was identified as a target of (20S) G-Rh2 in liver cancer cells [36]. Therefore, (20S) G-Rh2 holds promise as a multi-target drug for cancer therapy. In addition, we found other ginsenosides such as ginsenoside Rk1 and ginsenoside Rg5 also had strong inhibitory effects on cancer cells [59], but the anti-tumor mechanisms of these natural compounds remained unclear. Whether these ginsenosides exert their anti-tumor effect by targeting HSP90A or other proteins will be further investigated in our future studies.

## 4. Materials and Methods

### 4.1. Reagents and Plasmids

(20S) G-Rh2 (Sigma-Aldrich, St. Louis, MO, USA) was dissolved in 75% ethanol to a final concentration of 10 mM. MG-132 (MCE, Monmouth Junction, NJ, USA) was dissolved in dimethyl sulfoxide (DMSO) to a final concentration of 20 mM. NH_4_Cl (Sigma-Aldrich, St. Louis, MO, USA) was dissolved in phosphate buffered saline (PBS) to a final concentration of 2 M. MTT (Sigma-Aldrich, St. Louis, MO, USA) was dissolved in PBS to a final concentration of 5 mg/mL. Dulbecco modified Eagle’s medium (DMEM) was purchased from Gibco BRL (Gibco BRL, Grand Island, NE, USA).

Antibodies for β-Actin (sc-8432), CDK2 (sc-6248), Cyclin A (sc-271682), and pT821/T826-Rb (sc-271930) were purchased from Santa Cruz Biotechnology (Dallas, TX, USA). Antibodies for GAPDH (60004-1-Ig), HSP90A (60318-1-Ig), Cdc37 (10218-1-AP), CDK4 (11026-1-AP), CDK6 (66278-1-Ig), Cyclin B (55005-1-AP), Cyclin D (60186-1-Ig), Cyclin E (11554-1-AP), p21 (10355-1-AP), and Rb (10048-2-Ig) were purchased from Proteintech (Proteintech Group Inc., Rosemont, IL, USA). Mouse anti His-tag (AE003) was purchased from ABclonal (ABclonal Technology Co. Ltd., Wuhan, China). LC3 A/B (4108S) was purchased from CST (Cell Signaling Technology Inc., Boston, MA, USA). HRP-conjugated goat anti-rabbit IgG (H + L) secondary antibody (31460) and HRP-conjugated goat anti-mouse IgG (H + L) secondary antibody (31430) were purchased from Invitrogen (Invitrogen, Grand Island, NY, USA).

We amplified the genes that encode human HSP90A_N (a truncated HSP90A with middle and C-terminal domain deleted), human HSP90A_MC (a truncated HSP90A with N-terminal domain deleted), human HSP90A_MC-R413V, human HSP90A_MC-Q454E, and human HSP90A_MC-R413V/Q454E, respectively, by polymerase chain reaction (PCR), followed by a recombination into pEXS-DH-His vector (a gift from Fei Sun, Institute of Biophysics of Chinese Academy of Sciences), respectively, for protein expression in vitro. Primer pairs for PCR are shown in Table S2.

### 4.2. Cell Lines and Culture

Human liver cancer cell line HepG2 (HB-8065, ATCC, Manassas, VA, USA) and SK-HEP-1 (HTB-52, ATCC, Manassas, VA, USA) were cultured in DMEM high glucose medium containing 10% (*v/v*) fetal bovine serum (BI, Belt Haemek, Israel) with 100 units/mL penicillin and 100 μg/mL streptomycin at 37 °C in a humidified 5% CO_2_ atmosphere.

### 4.3. Protein Mass Spectrometry Analysis

HepG2 cells were lysed in immunoprecipitation (IP) lysis buffer (Pierce, Rockford, IL, USA) supplemented with Protease Inhibitor Cocktail (Roche, Indianapolis, IN, USA) and 1 mM phenylmethanesulfonyl fluoride (PMSF) (Sigma-Aldrich, St. Louis, MO, USA). Cell lysis with 2 mg of total protein was mixed with PEGA resin (Millipore, Billerica, MA, USA) covalently conjugated with (20S/R) G-Rh2, and incubated at 4 °C for 12 h on a tube rotator. Next, we washed the PEGA resin twice with PBS, and mixed the resin with 20 μg/mL trypsin solution at 37 °C for 12 h. The label-free protein analysis was performed by liquid chromatography tandem mass spectrometry (LC-MS), and the protein information was obtained from the UniProt database (http://www.uniprot.org/, accessed on 12 July 2021) by sequence alignment.

### 4.4. Phage Display Screening

We performed phage display screening with the T7 Select Human Liver Tumor cDNA Phage Library (Millipore, Billerica, MA, USA) according to the manufacturer’s protocol as previously described [36].

### 4.5. Network Topology Analysis

We collected 1263 liver cancer-related genes from the GeneCards database (https://www.genecards.org/, accessed on 16 July 2021) and DisGeNET database (https://www.disgenet.org/, accessed on 16 July 2021) with relevance score >20, and took the intersection of (20S) G-Rh2 potential targets and liver cancer-related genes. Then, the interaction network of the overlapped targets was constructed and analyzed using Cytoscape software (version 3.6.0) according to Table S1.

### 4.6. Gene Ontology and Pathway Enrichment Analysis

The overlapped targets were submitted to DAVID (https://david.abcc.ncifcrf.gov/, accessed on 16 July 2021), and the functional annotation chart was performed for GO and KEGG pathway enrichment analysis. Then, the circos diagram was drawn using the OmicShare tools, a free online platform for data analysis (http://www.omicshare.com/tools, accessed on 15 August 2021).

### 4.7. Molecular Docking Analysis

We downloaded the crystal structure of HSP90A_N (PDB ID: 4BQG) and HSP90A_MC (PDB ID: 3Q6M) from the RCSB Protein Data Bank (http://www.rcsb.org/pdb, accessed on 11 November 2021). We downloaded the three-dimensional structure of (20S) G-Rh2 (PubChem CID: 119307) from the NCBI PubChem Compound database (http://www.ncbi.nlm.nih.gov/pccompound, accessed on 11 November 2021). The structure of (20S) G-Rh2 was optimized by Chem3D (version 16.0, PerkinElmer, Waltham, MA, USA) to minimize its energy. Then, we performed molecular docking with AutoDock tools (version 4.2.6) with the default setting, based on the Lamarckian genetic algorithm (Scripps Research Institute, La Jolla, CA, USA). Results were optimized according to the empirical scoring function, which estimates the binding free energy of the predicted ligand–receptor complex, and shown by Discovery Studio 4.0 Visualizer (BIOVIA, Paris, France).

### 4.8. Prokaryotic Expression of the Truncated HSP90A In Vitro

The *E. coli* expression strain, BL21 (DE3), was transformed with pEXS-DH-HSP90A_N/HSP90A_MC (His-tagged construct), and cultured in Luria-Bertani (LB) liquid medium with 50 μg/mL ampicillin at 37 °C until the density reached an OD_600_ of 1.5. Then, the bacteria were cooled down to 16 °C and cultured for another 12 h at 16 °C with 1 mM isopropyl-beta-D-thiogalactopyranoside (IPTG) (Sigma-Aldrich, St. Louis, MO, USA) for protein expression.

### 4.9. Thermal Shift Assay

Cellular thermal shift assay (CETSA): HepG2 cells were seeded into 100 mm cell culture dishes with a cell density of 1 × 10^7^ cells per dish. Then, the cells were treated with 10 μM (20S)G-Rh2 at 37 °C for 1 h, and the control cells were incubated with the same volume of 75% ethanol. The treated cells were harvested, centrifugated at 3000× *g* for 5 min, and then resuspended in 1 mL PBS. The cell suspension was divided into eight aliquots, and heated with a thermal gradient from 40 °C to 100 °C for 4 min. After three repeated freeze–thaws with liquid nitrogen, the supernatant was separated by centrifugation at 20,000× *g* for 10 min. Then, 20 μL of each supernatant sample was analyzed by sodium dodecyl sulfate-polyacrylamide gel electrophoresis (SDS-PAGE) and immunoblot.

Iso-thermal dose response (ITDR): Prokaryotic-expressed HSP90A_N or HSP90A_MC with a concentration of 0.2 mM was mixed with the indicated concentrations of (20S)G-Rh2 from 0 μM to 10 μM, and then heated at 60 °C or 80 °C for 4 min. After centrifugation at 20,000× *g* for 10 min, 20 μL of each supernatant sample was used for SDS-PAGE and immunoblot analyses.

### 4.10. Co-Immunoprecipitation

A sample of 50 μL of Protein A/G Magnetic Beads (K0202, MCE, Monmouth Junction, NJ, USA) was balanced with 400 μL of IP lysis buffer three times. Five μg of primary antibody for immunoprecipitation was diluted with IP lysis buffer and rotated with the balanced beads for 2 h at 4 °C. Cell lysis with 500 μg of total protein was diluted to the final volume of 400 μL and then mixed with the above bead-antibody complexes, followed by another rotation for 2 h at 4 °C. The beads were then washed with IP lysis buffer three times and collected for immunoblot analyses.

### 4.11. Cell Cycle Analysis

HepG2 cells were treated with the indicated concentrations of (20S) G-Rh2 for 24 h at 37 °C in a humidified 5% CO_2_ atmosphere and were subsequently harvested and fixed with 70% ethanol at 4 °C for 12 h. After centrifugation at 1000× *g* for 5 min, the cells were resuspended with 1 mL PBS containing 50 μg/mL RNase A and incubated at 37 °C for 30 min. Then, 50 μg/mL PI (Beyotime Biotechnology, Shanghai, China) was used to stain the cells for another 30 min at 37 °C in a dark area. Red fluorescence was detected by flow cytometry (Beckman Coulter Life Sciences, Brea, CA, USA) at an excitation wavelength of 488 nm. The distribution of cell cycle phases was analyzed by ModFit LT 5.0 (Verity Software House, Topsham, ME, USA).

### 4.12. Cell Viability Assay

Exponentially growing HepG2 cells or SK-HEP-1 cells were seeded into a 96-well plate at 1 × 10^4^ cells per well. After incubation for 24 h, the cells were treated with increasing concentrations of (20S) G-Rh2 from 0 to 32 μM for 48 h. At the end of treatment, 20 μL of MTT (5 mg/mL) was added to each well and the cells were incubated for another 4 h. Then, the culture medium was removed, and the formazan grains formed by viable cells were solubilized with 150 μL DMSO. Absorption at 550 nm of each well was measured using a microplate reader (TECAN, Maennedorf, Switzerland).

### 4.13. Quantitative Real-Time Polymerase Chain Reaction (qRT-PCR)

Whole-cell RNA was isolated with TRIzol (Invitrogen, Grand Island, NY, USA), and 5 μg total RNA was used for cDNA synthesis with High-Capacity cDNA Reverse Transcription Kit (4368814, Applied Biosystems, Foster, CA, USA). Then, qRT-PCR analysis was performed on 7500 Real-time PCR system (Applied Biosystems, Foster, CA, USA) with PowerUp SYBR Green Master Mix (A25742, Applied Biosystems, Foster, CA, USA), followed by a three-step amplification program (one cycle at 95 °C for 30 s, followed by 45 cycles at 95 °C for 5 s, 50 °C for 15 s, and 72 °C for 10 s, with fluorescence signal collecting during the extension step of each cycle). Primer pairs were shown in Table S3. The gene expression level of CDKs was normalized to that of β-Actin.

### 4.14. Statistical Analysis

All data were obtained from independent triple-replicated experiments and shown as the mean ± standard deviation (SD). Significance was determined by a two-tail Student’s *t*-test using SPSS v18.0 (Chicago, IL, USA) as indicated (* *p* < 0.01, ** *p* < 0.001).

## 5. Conclusions

We found (20S) G-Rh2 shared 25 potential targets associated with liver cancer using bioinformatics analysis. The mass spectrometry and phage display results showed that HSP90A may serve as a key target of (20S) G-Rh2 in the treatment of liver cancer. Then, we proved the interaction between HSP90A and (20S) G-Rh2 by molecular docking and the thermal shift assay. The co-immunoprecipitation experiments were performed to validate the docking result. As shown in Figure 5D, (20S) G-Rh2 inhibited the HSP90A-Cdc37 PPI through binding to the N-terminal domain and the middle domain of HSP90A, inhibiting the maturation of CDKs to induce their UPS degradation and the anti-proliferation effect of HepG2 cells and SK-HEP-1 cells. In this study, we elucidated the anti-tumor mechanism of (20S) G-Rh2 by targeting the HSP90A-Cdc37 system in human liver cancer cells.

## Figures and Tables

**Table 1:** Molecular docking results of different domains of HSP90A with (20S) G-Rh2.

Result	Estimated Free Energy of Binding (kcal/mol)	Estimated Inhibition Constant, Ki (298.15 K)
4BQG:(20S) G-Rh2 result 1	−7.25	4.89 μM
4BQG:(20S) G-Rh2 result 2	−5.01	212.28 μM
3Q6M:(20S) G-Rh2	−6.32	23.18 μM

## Data Availability

Not available.

## Supplementary Materials

The following are available online at www.mdpi.com/article/10.3390/ijms222313170/s1.
