# Supplementary material for: (20S) Ginsenoside Rh2 Exerts Its Anti-Tumor Effect by Disrupting the HSP90A-Cdc37 System in Human Liver Cancer Cells
Source: Int J Mol Sci. 2021 Dec 6;22(23):13170. doi: 10.3390/ijms222313170 (PMC8658384; doi:10.3390/ijms222313170)
Supplement: Supplementary file 1 [file ijms-22-13170-s001.zip › ijms-1481641-supplementary.pdf]

Supplementary materials

Table S1. The network topology analysis of the overlapped genes between liver cancer related genes and (20S)G-Rh2 target proteins. Three topological indicators including degree of distribution, betweenness centrality, and closeness centrality represent the importance of these 25 genes in the background network of human genes. "Ratio R/S" represents the propensity of these 25 potential targets to bind (20S)G-Rh2 based on the mass spectrometry data, with a smaller ratio value representing a greater tendency to bind (20S)G-Rh2.

Gene	Degree	Betweenness Centrality	Closeness Centrality	Ratio R/S
STAT3	108	0.17989041	0.42120344	0.943045
HSP90AA1	91	0.06761578	0.4091858	0.565545
TRIP13	83	0.15713539	0.37716485	0.726409
HSPB1	44	0.04918154	0.36865204	0.732591
SFN	42	0.05349587	0.37837838	0.905909
ACTB	40	0.03128229	0.38181818	0.956295
KRT18	34	0.02438184	0.37191651	0.791659
NPM1	33	0.04089923	0.34406085	0.684182
HSPA5	31	0.03642254	0.34690265	0.401864
ITGB1	20	0.02094208	0.32272228	0.681409
KRT8	15	0.00602339	0.33295583	0.742864
HSPA9	14	0.01784429	0.3442623	0.447409
MSH2	13	0.00579711	0.31160572	0.513909
HSPA4	12	0.00944665	0.34854772	0.553795
PHB	12	0.00667437	0.33220339	0.595886
HSP90B1	10	0.0028511	0.30561331	0.739227
ANXA2	6	0.0013638	0.30753138	0.577295
IQGAP1	6	0.00076746	0.30371901	0.605591
COX5A	6	0.00552004	0.28201439	0.918659
CD44	5	0.00379146	0.28543689	0.602045
FASN	4	0.00403451	0.27735849	0.355182
MSH6	4	0.00027764	0.28296439	0.837250
BSG	2	0.00010345	0.2527945	0.270114
LRRC59	2	0.00006077	0.26320501	0.996250
PC	0	0	0	0.766182

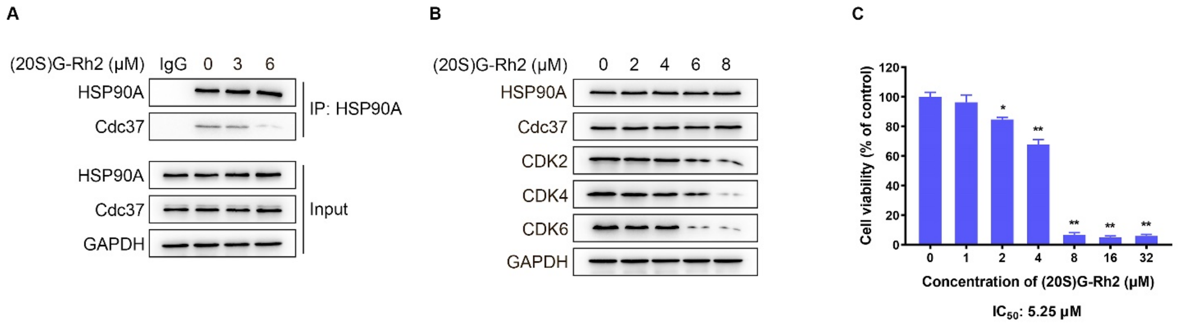

Figure S1. (20S)G-Rh2-induced interaction disruption of HSP90A and Cdc37, degradation of CDKs, and anti-proliferation effect of SK-HEP-1 cells. (A) Immunoprecipitation was performed with whole-cell lysate under treatment with the indicated concentrations of (20S)G-Rh2 for 24 h, and the interaction was analyzed by an immunoblot. (B) Protein levels of CDKs in SK-HEP-1 cells treated with (20S)G-Rh2 were determined by an immunoblot. (C) SK-HEP-1 cells were treated with the indicated concentrations of (20S)G-Rh2 for 48 h, and the cell viability was determined by MTT assay. All data are shown as the mean ± SD of experiments performed in triplicate. A two-tail Student's *t*-test was used for statistical analyses (*$p < 0.01$, **$p < 0.001$).

Table S2. Primers for vector construction.

Primer Description	Sequence
HSP90A_N-F	5′- CGGGATCCATGGAGGAGGAGGAGGTTGAGAC -3′
HSP90A_N-R	5′- GCCTCGAGATTTCAGGTTTGTCTTCCGACTCTTTC -3′
HSP90A_MC-F	5′- CGGGATCCGAAGAGCTCAACAAAACAAAGCCCATC -3′
HSP90A_MC-R	5′- GCCTCGAGATAAGGGGTGGCATTTCTTCAGTTACAG -3′
MC_R413V-F	5′- GCAAAATTTTGAAAGTTATCGTTAAGAATTTGGTCAAAAAATGC -3′
MC_R413V-R	5′- GCATTTTTTGACCAAATTCTTAACGATAACTTTCAAAATTTTGC -3′
MC_Q454E-F	5′- CTTGGAATACACGAAGACTCTGAAAATCGGAAGAAG -3′
MC_Q454E-R	5′- CTTCTTCCGATTTTCAGAGTCTTCGTGTATTCCAAG -3′

Table S3. Primers for qRT-PCR.

Gene Name	Sequence
CDK2-F	5′- ATGGATGCCTCTGCTCTCACTG -3′
CDK2-R	5′- CCCGATGAGAATGGCAGAAAGC -3′
CDK4-F	5′- CCATCAGCACAGTTCGTGAGGT -3′
CDK4-R	5′- TCAGTTCGGGATGTGGCACAGA -3′
CDK6-F	5′- GGATAAAGTTCCAGAGCCTGGAG -3′
CDK6-R	5′- GCGATGCACTACTCGGTGTGAA -3′
ACTB-F	5′- CACCATTGGCAATGAGCGGTTC -3′
ACTB-R	5′- AGGTCTTTGCGGATGTCCACGT -3′
